# The effect-site concentration of remifentanil blunting endotracheal intubation responses during anesthesia induction with etomidate: a dose-finding study

**DOI:** 10.1186/s12871-023-02165-2

**Published:** 2023-06-28

**Authors:** Zhencong Jiang, Jun Xiao, Xiaoqing Wang, Tao Luo

**Affiliations:** 1 Department of Anesthesiology, Peking University Shenzhen Hospital, Shenzhen, Guangdong province China; 2 Shantou University Medical College, Shantou, China

**Keywords:** Remifentanil, Etomidate, Endotracheal intubation, Effect-site concentration

## Abstract

**Background:**

Remifentanil can inhibit the hemodynamic responses caused by endotracheal intubation, but the effect-site concentration of it required to control intubation responses when combined with etomidate has not been demonstrated. The purpose of this study was to determine the effect-site concentration of remifentanil blunting tracheal intubation responses in 50% and 95% of patients (EC_50_ and EC_95_) during etomidate anesthesia.

**Methods:**

American Society of Anesthesiologists physical status (ASA) I-II elective surgical patients receiving target-controlled infusion (TCI) of remifentanil, followed by etomidate and rocuronium for anesthesia were enrolled. The Belive Drive A2 monitor was used to calculate the MGRSSI (Maygreen Sedative state index) of hypnotic effect and the MGRNOX (Maygreen Nociception index) of nociception. The MGRSSI and the MGRNOX value were generated every 1 s. Mean arterial pressure (MAP) and heart rate (HR) were measured every minute, noninvasively. Using the modified Dixon’s up-and-down method, the concentration of remifentanil was determined based on the intubation response of the previous patient. The cardiovascular response during endotracheal intubation was defined as positive when MAP or HR is 20% higher than the pre-intubation value. A probit analysis was used for calculating EC_50_, EC_95_ and 95% confidence interval (CI).

**Results:**

The EC_50_ and EC_95_ of remifentanil blunting tracheal intubation responses were found to be 7.731 ng/ml (95%CI: 7.212–8.278 ng/ml) and 8.701 ng/ml (95%CI: 8.199–11.834 ng/ml). There were statistically significant increases in HR, MGRSSI and MGRNOX value to tracheal intubation in the positive responses group compared to the negative group. The most common adverse event was postoperative nausea and vomiting, which occurred in 3 patients.

**Conclusion:**

Remifentanil effect-site concentration of 7.731 ng/ml is effective in blunting sympathetic responses to tracheal intubation in 50% of patients when combined with etomidate anesthesia.

**Trial registration:**

The trial was registered at the Chinese Clinical Trials Registry (www.chictr.org.cn, registration number: ChiCTR2100054565, date of registration: 20/12/2021).

## Background

In general anesthesia, endotracheal intubation is essential to protect the airway and assist in ventilation. At the same time, it can cause cardiovascular responses, including hypertension and tachycardia. A significant rise in catecholamine level occurs as a result of sympathetic excitation caused by parapharyngeal stimulations [1, 2]. These responses of endotracheal intubation generally have no significant impact on patients with normal circulatory system. However, they can be fatal to patients suffering from coronary heart disease, aneurysm, hypertension or other diseases [3].

Etomidate is commonly used as an anesthesia-inducing agent. It has the advantage of rapid onset, minor side effect on the cardiovascular system and respiratory system as well as few histamine release [4]. Nevertheless, the inhibitory effect of etomidate on laryngopharyngeal reflex is insufficient. Consequently, hypertension and tachycardia are frequent after intubation with etomidate anesthesia [5].

In order to attenuate the hemodynamic changes during intubation, several drugs in varied doses and by different routes have been investigated. Among them, the inhibitory effect of opioids on intubation responses has been confirmed [6–8]. Remifentanil is a selective µ-opioid receptor agonist that has rapid onset, short latency and short blood-effect-site equilibration time. Neither renal nor liver is affected by its metabolism, since it can be metabolized by nonspecific plasma and tissue esterase [9, 10]. Remifentanil is able to inhibit the activity of the sympathetic nervous system and enhancing anesthesia depth [11]. The cardiovascular responses to endotracheal intubation were blunted more effectively by remifentanil compared to fentanyl and sufentanil [12]. Etomidate in combination with remifentanil is frequently administered for anesthesia induction in a large number of surgical patients. Nevertheless, it remains unknown what is the proper concentration of remifentanil to control intubation responses.

**Fig. 1:**
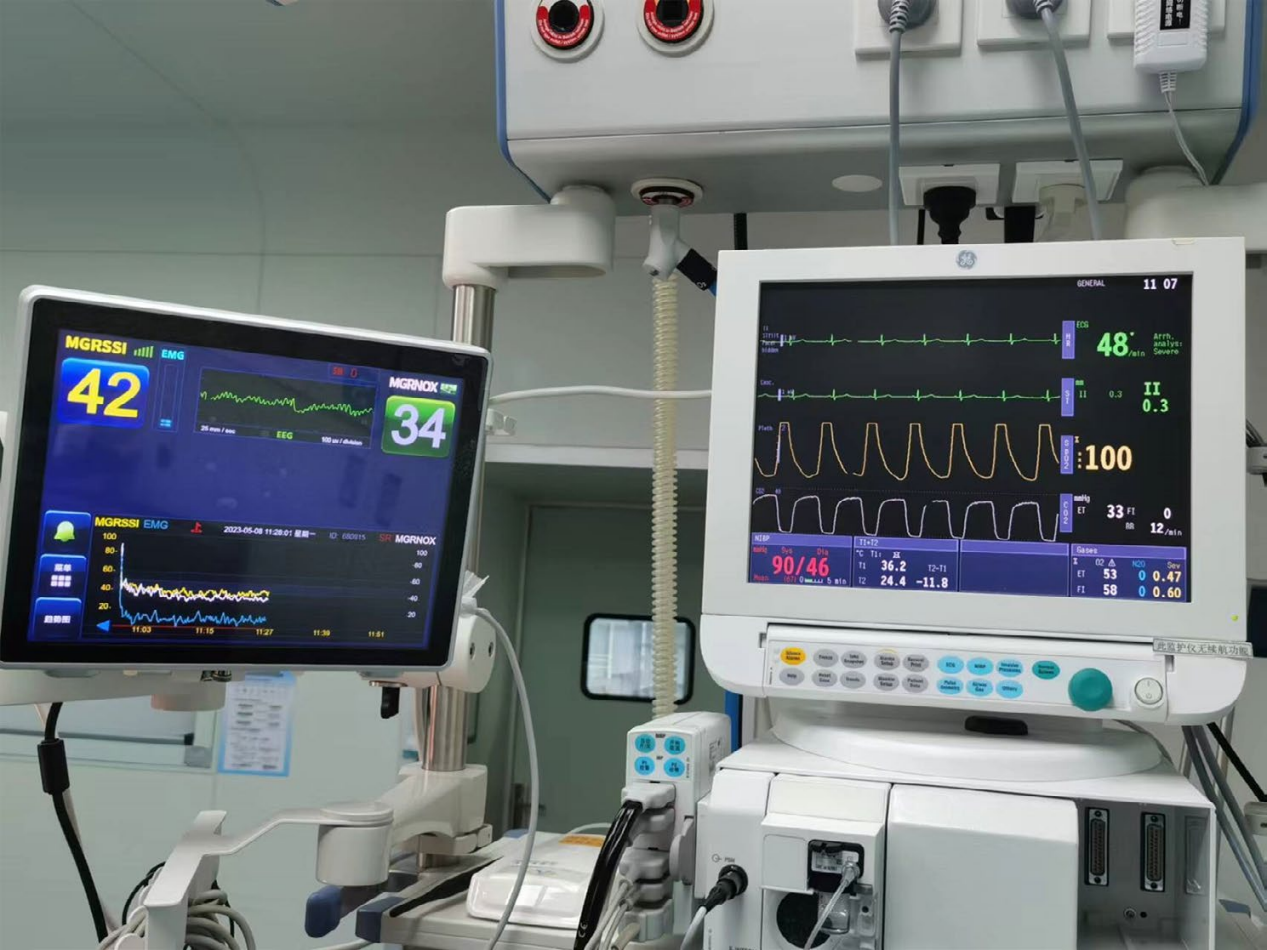
The Belive Drive A2 monitor (Maygreen, China)

The bispectral index (BIS) is most widely used to evaluate sedation level during general anesthesia [13]. However, its efficacy as an index of the anti-noxious component of anesthesia remains controversial [14, 15], as well as its accuracy in prediction the depth of anesthesia [16, 17]. In the present study, the Belive Drive A2 monitor (Maygreen, China) was used, which defines the MGRSSI of hypnotic effect and the MGRNOX of nociception. Figure 1 is a photo of the Belive Drive A2 monitor. A previous investigation has already validated hypnotic effect of the monitor [18], whereas nociception for general anesthesia has not been explored.

Therefore, the purpose of this study was to assess the effect-site concentration of remifentanil required to blunt sympathetic responses to tracheal intubation during etomidate anesthesia. We also examined whether changes in MGRSSI and MGRNOX may be adequate indices during painful stimuli such as laryngoscopy and intubation.

## Methods

### Inclusion and exclusion criteria

This prospective, double-blind, dose-finding clinical trial was approved by the Ethics Committee of the Peking University Shenzhen Hospital (approval number: 2021 No.096, date of approval: 06/12/2021) and registered at the Chinese Clinical Trial Registry (registration number: ChiCTR2100054565, date of registration: 20/12/2021). We obtained written informed consent from all enrolled patients.

ASA I-II patients who were 18 to 64 years old, with a body mass index between 18.5 and 30 kg/m^2^, and scheduled for general anesthesia for elective surgery (Otorhinolaryngology or gynecology) were approached to participate in this trial from January 2022 to March 2022. Patients were excluded if they (1) suffered from severe cardiovascular or respiratory diseases; (2) had renal, liver, or hematological dysfunctions; (3) diagnosed with obstructive sleep apnea-hypopnea syndrome or once had difficulty intubating; (4) had high risk factors of aspiration or regurgitation; (5) were taking beta-blockers, analgesics or psychotropic medications; (6) had contraindications to use remifentanil.

### Study protocol

All patients were fasted overnight and received no premedication prior to surgery. One of the arms was inserted with a venous catheter of 20- or 22-gauge after entering the operating room. During the perioperative period, electrocardiograms (ECG), pulse oximetry (SpO_2_), noninvasive blood pressure (NIBP) and end-tidal carbon dioxide (P_et_CO_2_) were monitored (N15 Anesthesia Monitor, Mindray, China). The MGRSSI and MGRNOX were also continuously monitored using electroencephalogram (EEG) derivative collected from forehead sensor (Belive Drive A2 Monitor, Maygreen, China).

The MGRSSI was recorded to assess the hypnotic effect of the anesthesia, while the MGRNOX was recorded to assess the nociception/antinociception balance. The MGRSSI and MGRNOX indices are based on calculation of the Fast Fourier Transform (FFT) and the Burst Suppression (BSR) that are fed into a regressive model which generates the output on a 0–100 scale. According to the manufacture’s recommendation, adequate sedation is typically represented by a MGRSSI range between 40 and 60, and adequate analgesia is represented by a MGRNOX range between 30 and 50.

Preoxygenation of patients with 100% oxygen via facial masks for 3 min was applied before intravenous induction. Anesthesia was performed using a TCI for administering remifentanil, which was started with a predetermined target effect-site concentration and infused according to Minto model [19], by an infusion pump (Fresenius Kabi, France). Two min after the remifentanil infusion began, etomidate (0.3 mg/kg) was injected intravenously within 30 s. When the patients lost consciousness (failed to respond to verbal commands), rocuronium (0.6 mg/kg) was given within 30 s, and artificial ventilation was initiated. A laryngoscopy was performed and endotracheal intubation was attempted 2 min after rocuronium injections, using a unified visual laryngoscope (E.An IIL, Tianjin Medan Medical corporation, China) and an ordinary endotracheal tube, the diameter of the tube was individualized by the patient’s height. All intubations were performed by one experienced attending anesthesiologist, those patients whose endotracheal intubation was not successful at one time or whose intubation time was longer than 1 min were excluded from the study. General anesthesia was maintained using 2% sevoflurane with 50% oxygen, end-tidal carbon dioxide concentrations were maintained at 35–45 mmHg using mechanical ventilation. Three min after endotracheal intubation, remifentanil’s effect-site concentration remained unchanged.

Systolic blood pressure (SBP), diastolic blood pressure (DBP), MAP, HR, SpO_2_, MGRSSI and MGRNOX were recorded 1 min before induction, 2 min and 1 min before intubation, at intubation and at each min for 3 min after intubation. Time 1 (T_1_) was defined as 1 min before induction and Time 2 (T_2_) was defined as 1 min before intubation. The pre-intubation values of MAP and HR were calculated as the mean of measurements taken 2 min and 1 min before intubation. The changes of MAP, HR, MGRSSI and MGRNOX during intubation (ΔMAP, ΔHR, ΔMGRSSI and ΔMGRNOX) were defined as the difference between the value 1 min before intubation and the maximal value within the first 3 min following intubation.

A modified Dixon’s up-and-down method was used to calculate the concentration of remifentanil [20–22]. Based on a previous study [15], remifentanil was administered at an effect-site concentration of 6 ng/ml to the first patient. If the cardiovascular response during endotracheal intubation (intubation response) was positive (MAP or HR is 20% higher than the pre-intubation value), the concentration of remifentanil in the next patient would decrease by 1 ng/ml. Conversely, if the intubation response was negative, the concentration of remifentanil would increase by 1 ng/ml. The modified up-and-down method we used was based on modifying the test space, so as to improve the accuracy of final results [21]. Specifically, following the first three “negative-positive” crossovers, the step change of dose was reduced to 0.5 ng/ml, this process was repeated until seven crossover points had been obtained.

During the data collection period, excessive hemodynamic fluctuations including: SBP <​ 80 or >​ 180 mmHg; HR <​ 50 bpm or > 120 bpm. Hypoxemia was defined as SpO_2_ ≤ 92% for more than 10 s. If the patients experienced excessive hemodynamic fluctuations, hypoxemia, severe muscle tremor, or persistent chest wall rigidity, we would handle it according to the emergency disposal plan and the patients were withdrawn from this study, the following case was treated with the same concentration of remifentanil.

### Blinding

The patients and the anesthesiologist for endotracheal intubation were not aware of the dose of remifentanil. An assistant anesthesiologist was responsible for administering remifentanil, recording various data and calculating the dose of remifentanil according to the previous patient’s response.

### Outcome measures

The primary outcomes were effective concentration of remifentanil blunting cardiovascular responses of intubation during etomidate anesthesia in 50% (EC_50_) and 95% (EC_95_) of the study population. Following EC_50_ calculation, the data were further analyzed for secondary outcomes to compare those who were positive and negative for tracheal intubation responses. The secondary outcomes including: (1) The changes of the hemodynamic indices (ΔMAP and ΔHR) and indices derived from electroencephalogram (ΔMGRSSI and ΔMGRNOX) during endotracheal intubation; (2) adverse events (great hemodynamic change, hypoxemia, muscle tremor, symptoms of chest wall rigidity, choking cough, and postoperative nausea and vomiting) related to remifentanil combined with etomidate anesthesia.

### Statistical analysis

Statistical analysis was performed using SPSS version 25.0 software (IBM, Armonk, NY, USA). For normal distribution, the data are presented as the mean ± standard deviation, while for nonnormal distributions, the date are presented as median (interquartile range). A Shapiro-Wilk test was used to check for normal distribution of continuous variables. The t-test was used to compare parametric data. Categorical variables were analyzed by the Chi‑square test or Fisher’s exact test. Significant differences were defined as P value < 0.05 (two-tailed). In order to calculate sample size, we conducted a pretest, assuming a standard deviation (SD) of 0.74 ng/ml and a standard error of the mean (SEM) of 0.23 ng/ml. Following Dixon and Massey’s suggestion, sample size needed to estimate EC_50_ was derived by n = 2*(SD/SEM)^2^ [23]. Considering an attrition rate of 10%, sample size needed to be at least 23 patients. When fewer than seven “negative-positive” crossovers appeared during the up-and-down sequence, the number should be increased. Based on the modified Dixon’s up-and-down method data, we calculated the EC_50_ and EC_95_ using a probit analysis. Statistical results are presented as point estimates (95% CI). To assess goodness-of-fit, Pearson’s Chi-squared test was used (P value > 0.05 indicates good fit).

## Results

**Fig. 2:**
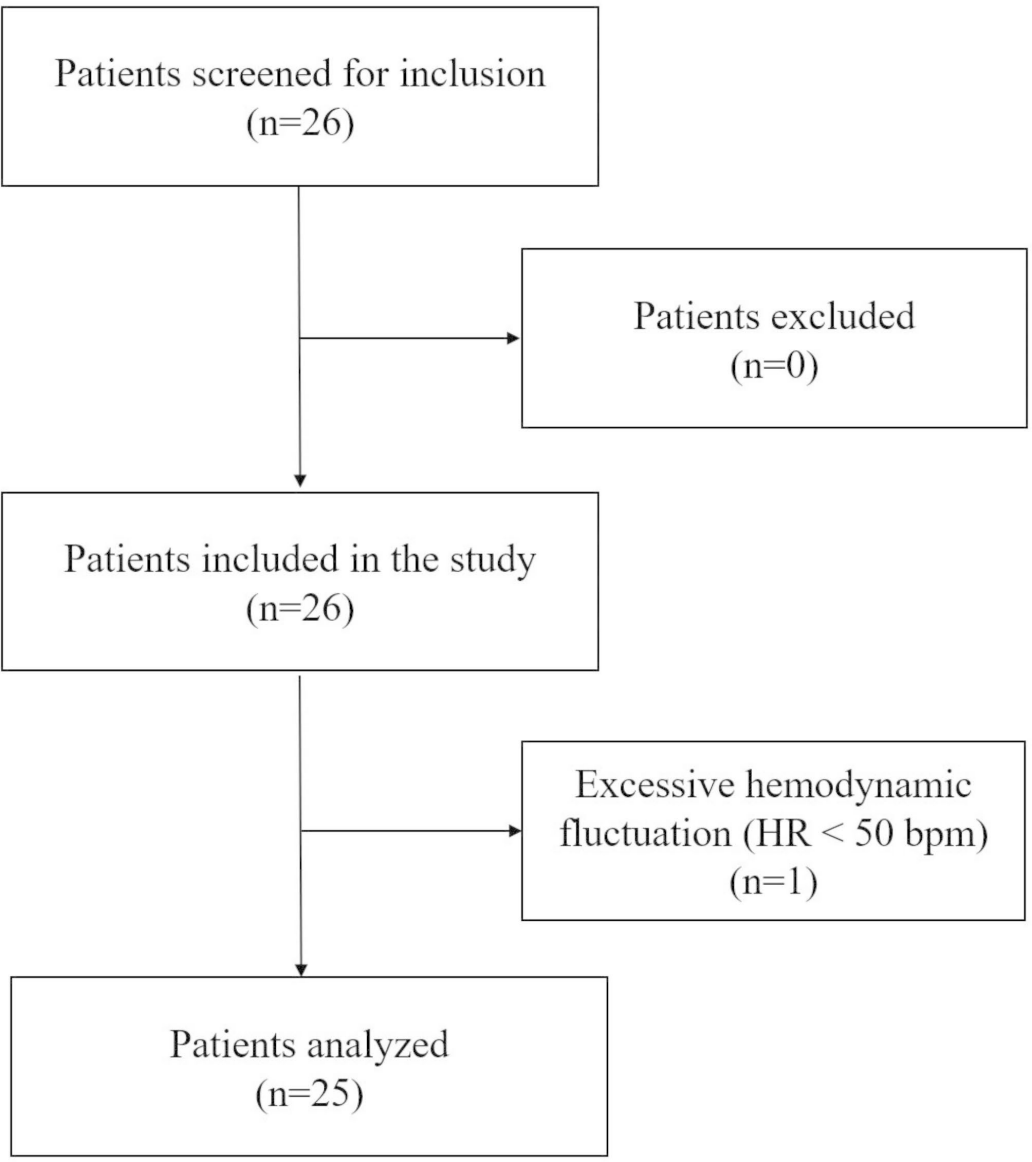
Study flow diagram

Twenty-six patients were screened for eligibility and all of them were recruited in the study. One patient was excluded from the study because of the bradycardia (HR < 50 bpm). Figure 2 illustrates the recruitment pathway. Table 1 shows the demographic data, induction profiles and surgical characteristics. According to the pre-defined intubation response, patients were divided into positive and negative group. There were no significant differences in patient characteristics (Table 1).

**Table 1:** Patient characteristics and clinical data

Characteristics	All patients (n = 25)	Positive group (n = 13)	Negative group (n = 12)	P value
Sex (male/female)	11/14	5/8	6/6	0.695
Age (years)	30.08 ± 5.90	29.77 ± 6.00	30.42 ± 6.04	0.791
Weight (kg) Height (cm) Body mass index (kg/m^2^) ASA physical status (I/II)	60.84 ± 11.27 164.60 ± 6.89 22.33 ± 3.03 20/5	59.54 ± 8.47 164.62 ± 7.12 21.90 ± 2.25 11/2	62.25 ± 13.96 164.58 ± 6.93 22.79 ± 3.74 9/3	0.559 0.991 0.478 0.645
Types of surgery				
Gynecology Otorhinolaryngology	3 22	2 11	1 11	--- ---
Mean arterial pressure (mmHg)				
T_1_ T_2_	84.28 ± 9.29 70.40 ± 8.91	85.38 ± 9.49 68.77 ± 7.99	83.08 ± 9.33 72.17 ± 9.84	0.547 0.352
Heart rate (bpm)				
T_1_ T_2_	75.84 ± 6.47 67.04 ± 8.03	76.54 ± 6.62 66.38 ± 5.98	75.08 ± 6.50 67.75 ± 10.04	0.585 0.680

Values are presented as mean ± SD or absolute numbers. ASA: American Society of Anesthesiologists. T_1_: 1 min before induction. T_2_: 1 min before intubation

**Fig. 3:**
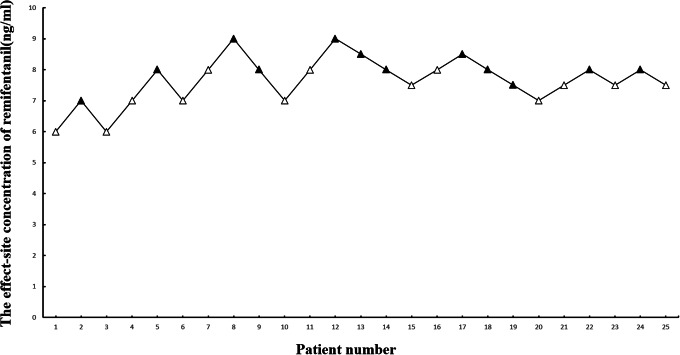
Individual responses to endotracheal intubation according to the up-and-down method. A higher effect-site concentration of remifentanil was given to the next patient when a patient showed positive hemodynamic response during endotracheal intubation (empty triangle). A lower effect-site concentration of remifentanil was given to the next patient when a patient showed negative hemodynamic response during endotracheal intubation (filled triangle)

**Fig. 4:**
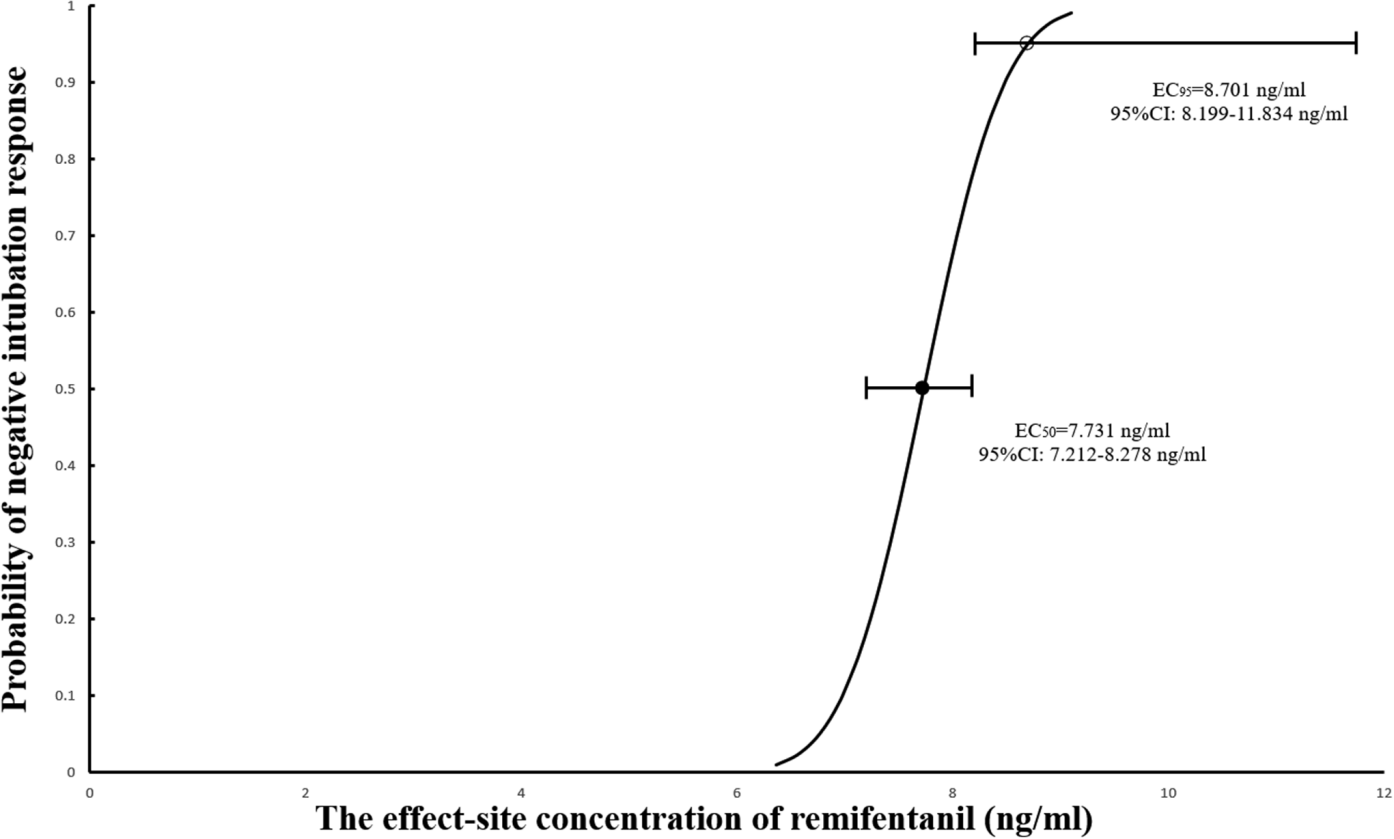
Dose–response curve of the effect-site concentration of remifentanil and negative intubation response. The black circle represented the 50% probability and the white circles represented the 95% probability

Figure 3 presents individual responses to endotracheal intubation according to the up-and-down method. The EC_50_ and EC_95_ of remifentanil blunting cardiovascular responses to intubation during etomidate anesthesia were found to be 7.731 ng/ml (95%CI: 7.212–8.278 ng/ml) and 8.701 ng/ml (95%CI: 8.199–11.834 ng/ml). Pearson’s Chi‑squared goodness‑of‑fit (χ^2^ = 1.177, P = 0.882). Figure 4 illustrates the dose-response curve of the remifentanil concentration and the probability that a negative intubation response would occur.

MAP and HR decreased gradually in both groups after remifentanil and etomidate administration (Table 1). ΔMAP, ΔHR, ΔMGRSSI and ΔMGRNOX during endotracheal intubation of two groups are presented in Table 2. The ΔHR (P < 0.001), ΔMGRSSI (P = 0.005) and ΔMGRNOX (P = 0.034) between the were significant higher in endotracheal intubation positive group than that of negative group. By contrast, no significant difference was observed between groups for ΔMAP (P = 0.091).

**Table 2:** The changes of the hemodynamic indices (ΔMAP and ΔHR) and indices derived from electroencephalogram (ΔMGRSSI and ΔMGRNOX) during endotracheal intubation

Variables	All patients (n = 25)	Positive group (n = 13)	Negative group (n = 12)	P value
ΔMAP (mmHg)	11.12 ± 7.68	13.62 ± 8.51	8.42 ± 5.85	0.091
ΔHR (bpm)	16.96 ± 8.41	22.62 ± 6.91	10.83 ± 4.86	< 0.001^**^
ΔMGRSSI ΔMGRNOX	15.60 ± 8.79 8.96 ± 6.06	20.08 ± 8.50 11.38 ± 6.33	10.75 ± 6.37 6.33 ± 4.68	0.005^**^ 0.034^*^

Values are presented as mean ± SD. * P < 0.05; ** P < 0.01

The most common adverse events observed in this study were postoperative nausea and vomiting (12% of the patients) and transient muscle tremor (8% of the patients). None of the patient showed hypoxemia, choking cough and chest wall rigidity (Table 3).

**Table 3:** Adverse events observed in this study

Adverse events	No. (%)
postoperative nausea and vomiting	3 (12.00%)
transient muscle tremor	2 (8.00%)
hypoxemia	0
choking cough	0
chest wall rigidity	0

Data are presented as absolute numbers (%)

## Discussion

Our results showed that achieving an 50% effect-site concentration of remifentanil at 7.731 ng/ml blunts hemodynamic responses to endotracheal intubation in patients receiving etomidate anesthesia.

Remifentanil as an adjuvant anesthetic drug, has been shown to effectively blunt the hemodynamic response to laryngoscopy and endotracheal intubation when administered by bolus or infusion. O’Hare et al. compared remifentanil 0.5, 1.0, or 1.25 µg/kg given as a bolus over 30 s, on pressor response to intubation during rapid sequence induction, they found that the dose of 0.5 µg/kg was ineffective [24]. The hemodynamic response to laryngoscopy and tracheal intubation could be inhibited when remifentanil 0.5 µg/kg was given over 30 s, followed by an infusion of 0.25 µg/kg/min over 3 min [25]. Higher bolus doses at 1.0 and 1.25 µg/kg were sufficient in controlling heart rate and arterial pressure increase after intubation. However, bolus injection of the 1.25 µg/kg caused adverse reactions such as hypotension and bradycardia [24]. The incidence of remifentanil-associated bradycardia and hypotension can be greatly reduced in the presence of a vagolytic agent [7].

Since a single bolus injection of large doses remifentanil can cause side effects such as bradycardia and hypotension, many studies have evaluated the efficacy of TCI of remifentanil on cardiovascular response to endotracheal intubation. Albertin et al. found that approximately 50% of patients with tracheal intubation show reduced cardiovascular responses when effect-site concentration of remifentanil of 5 ng/ml, combining with target-controlled infusion of propofol to maintain a BIS value ranging between 40 and 50 [15]. Similarly, the EC_50_ (± SD) value of remifentanil was 5.58 ± 0.75 ng/ml for orotracheal with propofol at a target effect-site concentration of 5.0 µg/ml [26]. When using Surgical Stress Index, a measurement of sympathetic cardiovascular responses based on heart rate and photoplethysmographic pulse wave amplitude as a target, Mustola et al. found that the effect-site concentrations of remifentanil attenuating intubation responses in 50% of patients was 3.05 ± 0.27 ng/ml when anesthesia state entropy was maintained between 40 and 60 [27].

Our results showed that the 50% effect-site concentration of remifentanil was 7.731 ng/ml. This is somewhat higher than previous reports. Several factors may affect the effect-site concentration of remifentanil to blunt intubation responses. First, different sedatives have different effect on the cardiovascular system. The use of propofol generally resulted in less hypertension and tachycardia during and after intubation [28]. In contrast, because etomidate is unable to prevent intubation responses, a higher effect-site concentration of remifentanil is needed to blunt the stress response after intubation when combined with etomidate. Second, the definitions of positive response to tracheal intubation vary from different studies. We used an increase of 20% or more in either heart rate or mean arterial pressure of the pre-intubation values as a positive response to tracheal intubation. Other investigations used surgical stress index, Surgical Pleth Index and tracheal intubation conditions as measurements of intubation responses [26, 27, 29]. In a previous study, although increase of 15% or more in heart rate or mean arterial pressure is defined as a positive intubation response, patients that unable to keep cardiovascular variables within 15% values before intubation were withdrawn from the study [15]. In our study, however, the hemodynamic parameter remained relative stable when TCI remifentanil combined with etomidate was used for anesthesia induction.

We found statistically significant increase in heart rate, MGRSSI and MGRNOX value to tracheal intubation but no change in mean arterial pressure in the positive responses group compared to the negative responses group. Since the primary outcomes of the study were effective concentration of remifentanil blunting cardiovascular responses to endotracheal intubation, it is difficult to make absolute assertions which of the measurements is more sensitive to predict intubation responses. In addition, since we used a non-continuous method of monitoring blood pressure with an interval of 1 min, it is difficult to make absolute evaluation about changes in this parameter. However, our results suggested that both MGRSSI and MGRNOX changes may be as sensitive as heart rate to nociceptive stimulation for detecting deficiencies in the anti-noxious component of anesthesia. Furthermore, inhalation of sevoflurane after intubation may affect the tracheal intubation responses. however, same doses of etomidate and sevoflurane for each patient were administer to control the confounding factors.

Conventionally, hemodynamic response is considered as the marker of noxious stimuli during surgery. Although Bispectral index is commonly used to monitor hypnotic state, it has the limitations in predicting hemodynamic responses to a painful stimuli such as laryngoscopy and endotracheal intubation [14, 15]. The bispectral index can also be affected by several different factors in addition to the hypnotic state [16, 17]. Nociception-induced changes in the EEG include beta arousal, delta arousal, and alpha dropout patterns [30]. Recognizing and quantifying these responses could help to optimize intraoperative anti-noxious management. Jensen et al. previously demonstrated that the qCON 2000 processed-EEG indices, a monitor of both hypnotic and analgesic effect of anesthesia, could predict the movement response to surgical stimuli [31]. However, whether its index change is as sensitive as hemodynamic responses was not explored. Our preliminary finding that both MGRSSI and MGRNOX changes were as sensitive as heart rate to trachea intubation, fosters further investigations to examine whether these indices are highly specific and sensitive indicator of hypnotic effects and of response to noxious stimulation.

## Conclusion

In summary, our study demonstrated that target-controlled infusion concentration of remifentanil at 7.731 ng/ml and 8.701 ng/ml blunt sympathetic responses in 50% and 95% of ASA I-II adult patients respectively, when combined with bolus infusion of etomidate at 0.3 mg/kg. Since etomidate is associated with a more stable hemodynamics compared with other anesthesia induction drugs such as propofol, our finding warrants future prospective study evaluating the efficacy and safety of target-controlled concentration of remifentanil with etomidate for anesthesia induction in more specific groups of patients, who are elderly or medically compromised.

## Data Availability

The datasets of this study are not publicly available because relevant policies of the Ethics Committee, but they are available from the corresponding author on reasonable request.

## Abbreviations

EC_50_ and EC_95_: Effective concentration in 50% and 95% of the study population
ASA: American Society of Anesthesiologists
TCI: Target-controlled infusion
MAP: Mean arterial pressure
HR: Heart rate
BIS: Bispectral index
MGRSSI: Maygreen Sedative state index
MGRNOX: Maygreen Nociception index
EEG: electroencephalogram
CI: Confidence interval
ECG: Electrocardiograms
SpO_2_: Pulse oximetry
NIBP: Noninvasive blood pressure
P_et_CO_2_: End-tidal carbon dioxide
SBP: Systolic blood pressure
DBP: Diastolic blood pressure
SD: Standard deviation
SEM: Standard error of the mean
FFT: Fast Fourier Transform
BSR: Burst Suppression.
